# Genipin alleviates vascular hyperpermeability following hemorrhagic shock by up-regulation of SIRT3/autophagy

**DOI:** 10.1038/s41420-018-0057-2

**Published:** 2018-05-09

**Authors:** Cai Shumin, Xu Wei, Li Yunfeng, Liang Jiangshui, Gao Youguang, Chen Zhongqing, Li Tao

**Affiliations:** 1 Department of Critical Care Medicine, Nanfang Hospital, Southern Medical University/The First School of Clinical Medicine, Southern Medical University, Guangzhou, 510515 China; 2 Department of Critical Care Medicine, The First People’s Hospital of Chenzhou/Institute of Translation Medicine, University of South China, Chenzhou, 423000 China; 3 Department of Lung Cancer Diagnosis and Treatment Center, The First People’s Hospital of Chenzhou/Institute of Translation Medicine, University of South China, Chenzhou, 423000; China; 4 Department of Anesthesiology, The First Affiliated Hospital of Fujian Medical University/The First School of Clinical Medicine, Fujian Medical University, 20 Chazhong Road, Fuzhou, 350005 China

## Abstract

Genipin (GP) is commonly used to treat cardiovascular diseases; however, the protective action of GP against vascular hyperpermeability (VH) has not been reported. We previously reported that intrinsic apoptotic signaling (IAS) is involved in VH following hemorrhagic shock (HS). GP inhibits apoptosis, but the specific mechanism remains unclear. In the present study, we observed that GP protects against HS-induced VH in vitro and in vivo. We report that this protective effect is related to the inhibition of IAS by up-regulation of autophagy via sirtuin 3 (SIRT3). The endothelial cell hyperpermeability induced by HS was enhanced by GP; this was attenuated by 3-methyladenine (3MA), a specific inhibitor of autophagy, indicating the involvement of autophagy. Consistent with these results, we found that 3MA reversed the effects of GP on up-regulation of autophagy, and also diminished the protective effect of GP against IAS activation following HS. Furthermore, knockout of SIRT3 inhibited GP-induced autophagy, indicating the requirement of SIRT3 in the regulation of autophagy by GP. In rats, GP improved HS-induced VH, which was repressed by 3MA and 3-(1*H*-1,2,3-triazol-4-yl)pyridine (3-TYP), a SIRT3 inhibitor. In conclusion, these findings suggest that autophagy plays a protective effect in VH following HS; the protective effect of autophagy is reinforced by GP, which protects against IAS and VH by up-regulating SIRT3.

## Introduction

Hemorrhagic shock (HS) has been implicated in the pathogenesis of multi-organ failure, and accounts for 30% of deaths associated with traumatic injury^1^. One of the primary clinical manifestations of HS is disruption of the endothelial cell barrier, which leads to vascular hyperpermeability (VH)^2^. However, the clinical treatment options for VH remain poor. Genipin (GP) is an aglycone derived from geniposide, an iridoid glycoside, and is a major component of the fruit of *Gardenia jasminoides*. GP has been used in the pharmacological treatment of cardiovascular disorders, but the protective effect of GP against VH as not been reported.

Autophagy is an intracellular self-degradation process in which double-membrane organelles, termed autophagosomes, deliver cytoplasmic materials to lysosomes^3^. It has been suggested that the autophagic machinery plays an important role in regulating endothelial permeability^4,5^, but the mechanism of this regulation has not been clarified. In a previous study, we suggested that intrinsic apoptotic signaling (IAS) is involved in HS induced by VH^6^. In addition, autophagy exerts its protective effects by preventing apoptosis^7,8^. In a recent study, it was demonstrated that GP promotes autophagy; however, the mechanism of action remains unclear.

Sirtuin proteins (SIRT) are a family of NAD-dependent protein deacetylases and/or ADP ribosylases that control metabolic homeostasis^9^. SIRT3 regulates autophagy via the deacetylation of several autophagy-related genes (ATGs), which perform important roles in autophagy^10–12^. In the present study, we investigated the protective effects of GP against VH during HS. In particular, we evaluated the induction of autophagy following up-regulation of SIRT3. Our present study may provide a novel strategy to counteract VH, which is a challenging problem in intensive care.

## Results

### GP mitigates H/R-induced PMVEC monolayer hyperpermeability in an autophagy-dependent manner

Pulmonary microvascular endothelial cells (PMVECs) were divided into four groups: control group (cells incubated in normoxic conditions without other treatment); hypoxia/reoxygenation (H/R) group (cells pretreated with the vehicle for GP or 3MA and then exposed to H/R); GP group (cells pretreated with 50 μM GP and then exposed to H/R); and 3MA group (cells pretreated with 5 mM 3MA and 50 µM GP and then exposured to H/R).

**Fig. 1:**
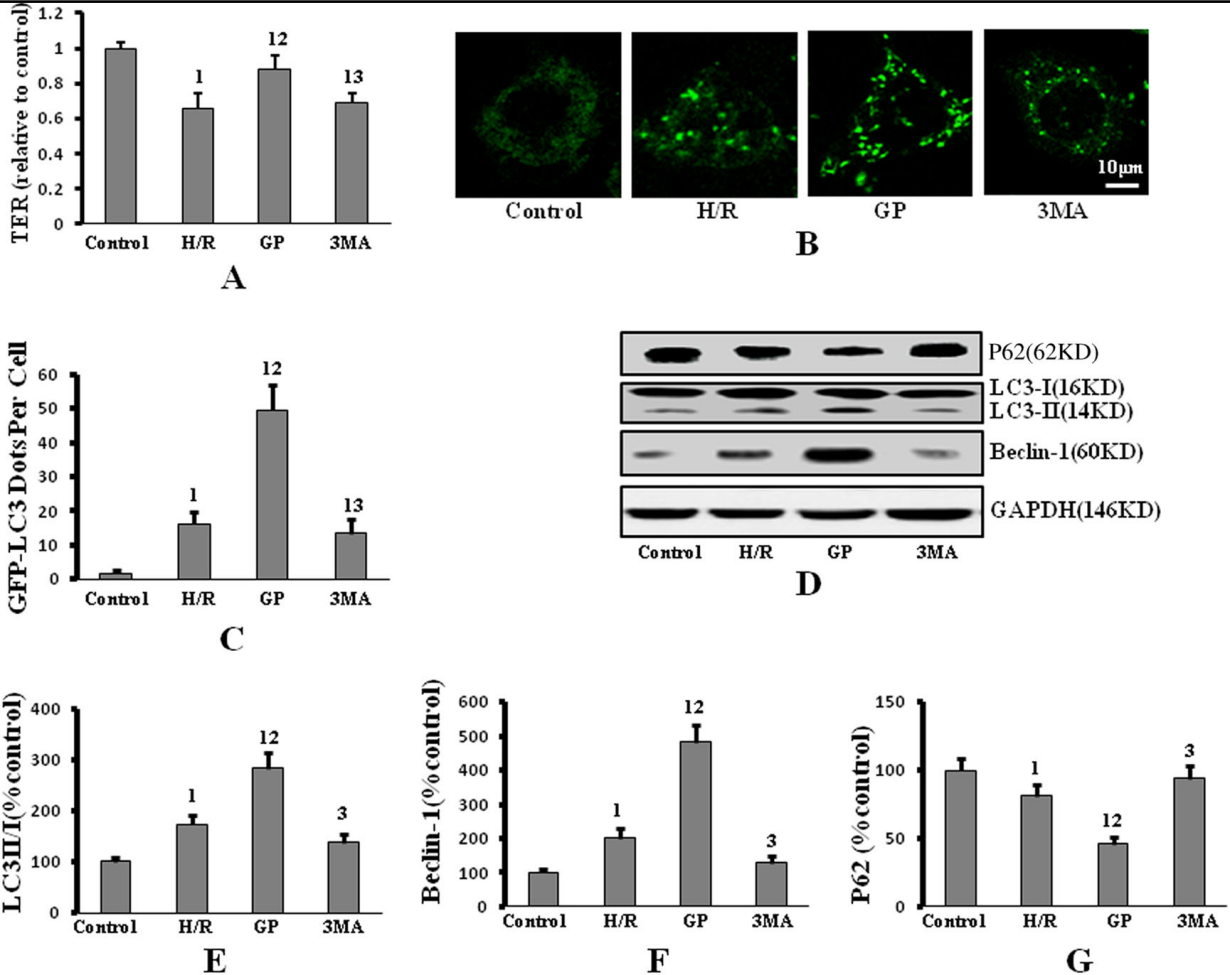
GP treatment improved H/R-induced monolayer hyperpermeability and up-regulated H/R-induced autophagy, which were depressed by 3MA. **a** Monolayer hyperpermeability was examined by detecting the TER of the PMVEC monolayer. **b** Cells were transfected with GFP-LC3, followed by exposure to H/R (or control); GFP-LC3 punctae formation was visualized by confocal microscopy (×650 magnification). **c** Quantitative analysis of the number of GFP-LC3 puncta-positive cells. **d** Expression of P62, LC3, and Beclin-1 protein was measured by western blot. **e** Quantification of LC3 II/I protein expression by densitometry. **f** Quantification of Beclin-1 protein expression by densitometry. **g** Quantification of P62 protein expression by densitometry. Data are presented as mean ± SD (*n* = 6 in each group). 1: *P* < 0.05 compared with control the group; 2: *P* < 0.05 compared with the H/R group; 3: *P* < 0.05 compared with the GP group

PMVEC monolayer hyperpermeability was examined by determining the transendothelial electrical resistance (TER) of the cell monolayer. H/R-induced monolayer hyperpermeability was indicated by a decrease in the TER of the cell monolayer in the H/R group compared to the control group (Fig. 1a). GP treatment mitigated the H/R-induced monolayer hyperpermeability compared to vehicle treatment alone (Fig. 1a). However, 3MA, an inhibitor of autophagy, depressed the protective effect of GP on H/R-induced monolayer hyperpermeability, as indicated by decreased TER of the cell monolayer in the 3MA group compared to the GP group; this demonstrates the involvement of autophagy in the protective action of GP against H/R injury.

The green fluorescent protein and LC3 fusion protein, GFP-LC3, provides a useful indicator of autophagy through the evaluation of LC3 punctae. When autophagy occurs, GFP-LC3 foci redistribute from a diffuse pattern to a punctate cytoplasmic pattern. GP treatment increased H/R-induced formation of GFP-LC3 punctae, which was depressed by 3MA treatment (Fig. 1b, c). These data indicate that GP up-regulates autophagy in PMVECs in response to H/R injury.

To investigate the effects of GP on autophagy, the expression levels of Beclin-1, P62, and LC3 were determined. Expression of LC3 II/I and Beclin-1 increased, and expression of P62 decreased, in PMVECs treated with vehicle compared to the control group, indicating the activation of autophagy in response to H/R injury (Fig. 1d–g). Moreover, GP treatment enhanced the expression of Beclin-1 and LC3 II, and decreased the expression of P62 as compared with H/R group; these changes were reversed by 3MA treatment.

### 3MA treatment reversed the protective effect of GP against H/R-induced intrinsic apoptotic signaling

To investigate whether GP attenuated H/R-induced intrinsic apoptotic signaling, mitochondrial dysfunction (MD) (mitochondrial membrane potential (MMP), mitochondrial permeability transition pore (mPTP), intracellular ROS level and cellular ATP), cytochrome *c* release, and caspase-3 activity were evaluated. PMVECs were divided into four treatment groups, as above.

**Fig. 2:**
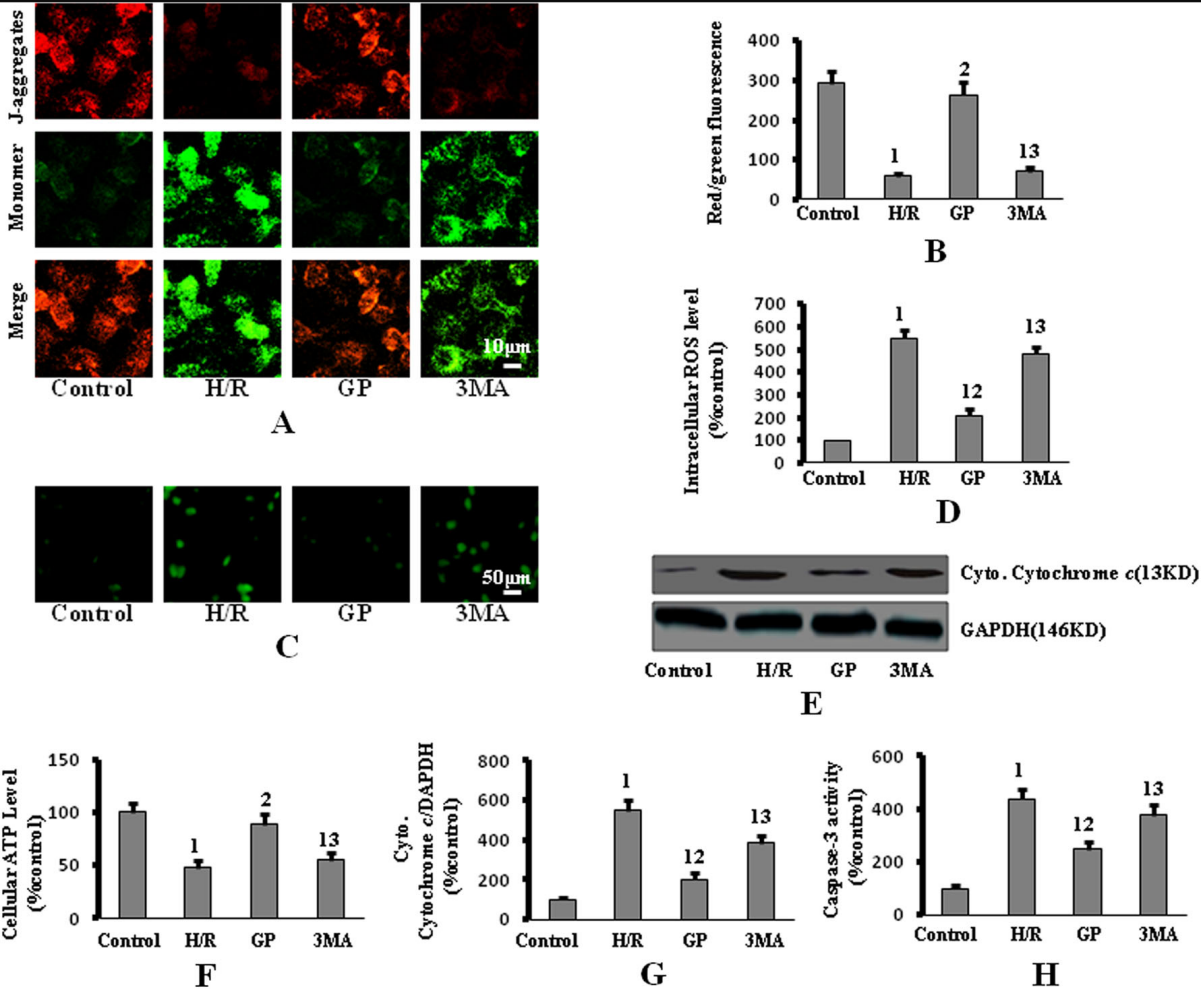
Treatment with 3MA reversed the protective effect of GP against H/R-induced IAS activation. **a** Intracellular red and green fluorescence of JC-1 was determined by fluorescent inverted microscopy (×400 magnification). **b** The intracellular red and green fluorescence of JC-1 was measured by flow cytometry. **c** Intracellular ROS levels were determined by DCFH-DA. **d** ROS levels were quantified by an automatic microplate reader. **e** Cytoplasmic cytochrome c was measured by western blot. **f** Intracellular ATP levels were determined by a luciferase-based assay. **g** Quantification of cytoplasmic cytochrome *c* protein expression by densitometry. **h** Caspase-3 activity was measured using a caspase-3 fluorometric assay kit. Data are presented as mean ± SD (*n* = 6 in each group). 1: *P* < 0.05 compared with the control group; 2: *P* < 0.05 compared with the H/R group; 3: *P* < 0.05 compared with the GP group

JC-1 (5,5′,6,6′-Tetrachloro-1,1′,3,3′-tetraethyl-imidacarbocyanine iodide), the potential-sensitive fluorescent dye, form aggregates in normally polarized mitochondria, and exists as monomers in damaged and depolarized mitochondria. The color of this dual-emission probe changes from red-orange to green as the mitochondrial membrane is depolarized. Control cells treated with JC-1 demonstrate red fluorescence, indicating normal mitochondria (Fig. 2a); however, H/R exposure rapidly caused MMP dissipation, indicated by an increase in green fluorescence and the simultaneous disappearance of red fluorescence (Fig. 2a). Treatment with GP significantly attenuated the H/R-induced changes in MMP, as indicated by the repression of green fluorescence and retention of red fluorescence. Moreover, the intracellular ATP levels were decreased and the ROS levels were increased in vehicle-treated PMVECs compared to the control group (Fig. 2c, d, f), while treatment with GP resulted in an increase in ATP levels and decrease in ROS levels. Depolarized mitochondria, increased ROS levels and lower ATP levels were observed in the 3MA treatment group compared to the GP treatment group, indicating that 3MA attenuates the improvement of H/R-induced MD following GP treatment.

IAS is activated through the mitochondrial release of cytochrome *c*, which releases the apoptosome assembly from apoptotic protease-activating factor 1, ATP, and procaspase-9, leading to morphologic and functional cellular alterations via the activation of caspase-3^2,6^. Levels of cytoplasmic cytochrome *c* and caspase-3 activity were increased in the H/R group compared to the control group; these indicators of apoptosis were depressed by treatment with GP (Fig. 2e, g, h). However, treatment with 3MA reversed the effects of GP, as evidenced by increased cytoplasmic cytochrome *c* and caspase-3 activity in the 3MA group compared with the GP group (Fig. 2e, g, h).

### SIRT3 is required for GP-induced autophagy and protection against intrinsic apoptotic signaling in H/R

SIRT3 plays important role in autophagy^10^. To confirm whether SIRT3 participates in GP-induced autophagy, we silenced SIRT3 in PMVECs with RNA interference (RNAi). PMVECs were divided into four groups: control group (cells transfected with a scrambled non-targeting small interfering RNA (siRNA) vector and then incubated in normoxic conditions); H/R group (cells pretreated with a non-targeting vector and then exposed to H/R); GP group (cells transfected with a non-targeting vector and pretreated with 50 µM GP and then exposed to H/R); SIRT3-knockdown group (cells transfected with SIRT3-targeting siRNA vector were treated with 50 µM GP and then exposed to H/R).

**Fig. 3:**
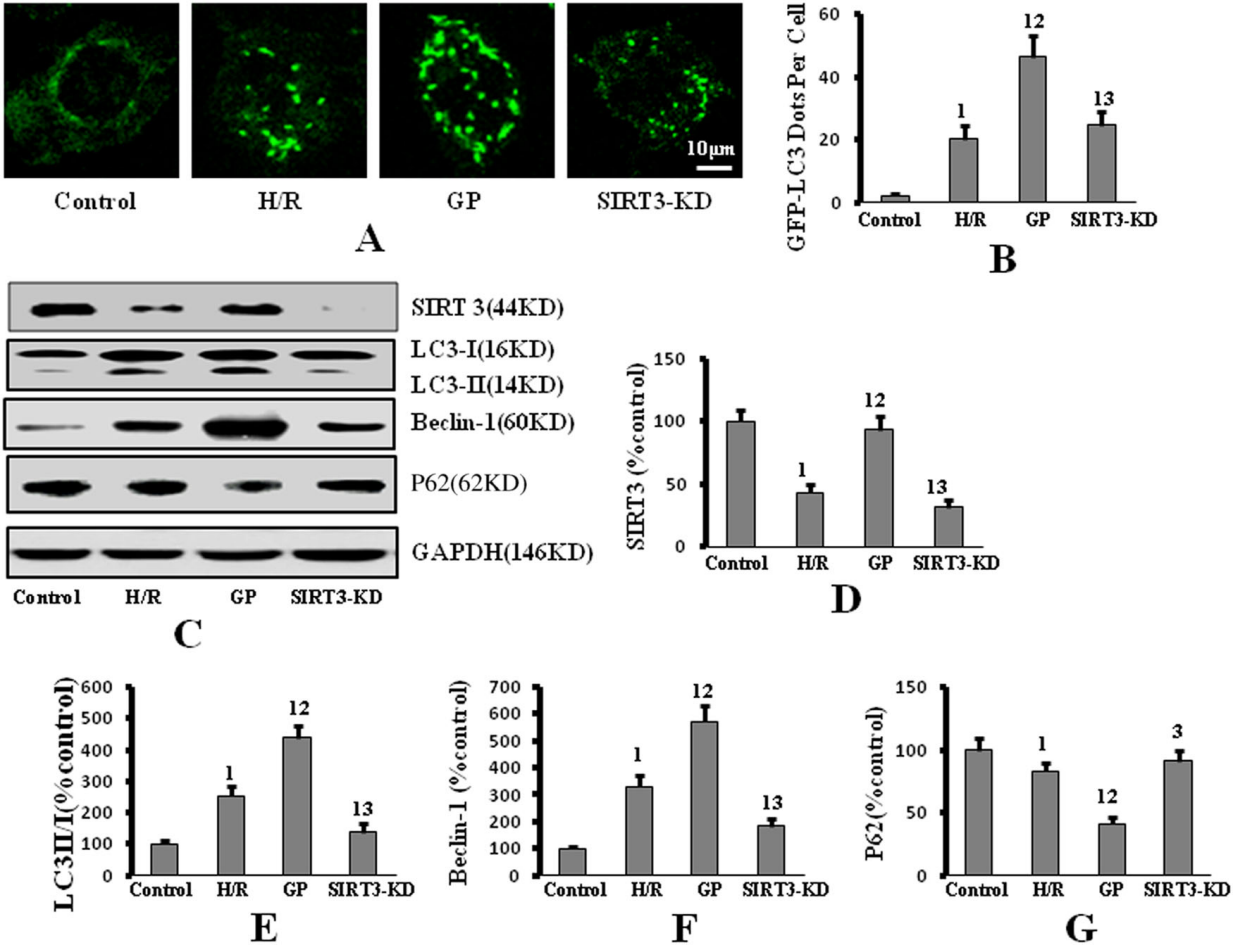
Silencing of SIRT3 repressed GP-induced autophagy following H/R. **a** Cells were transfected with GFP-LC3, followed by H/R (or control) exposure. GFP-LC3 punctae formation was visualized by confocal microscopy (×650 magnification). **b** Quantitative analysis of the number of GFP-LC3 puncta-positive cells. **c** P62, LC3, and Beclin-1 protein expression were measured by western blot. **d** Quantification of SIRT3 protein expression by densitometry. **e** Quantification of LC3 II/I protein expression by densitometry. **f** Quantification of Beclin-1 protein expression by densitometry. **g** Quantification of P62 protein expression by densitometry. Data are presented as mean ± SD (*n* = 6 in each group). 1: *P* < 0.05 compared with the control group; 2: *P* < 0.05 compared with the H/R group; 3: *P* < 0.05 compared with the GP group

**Fig. 4:**
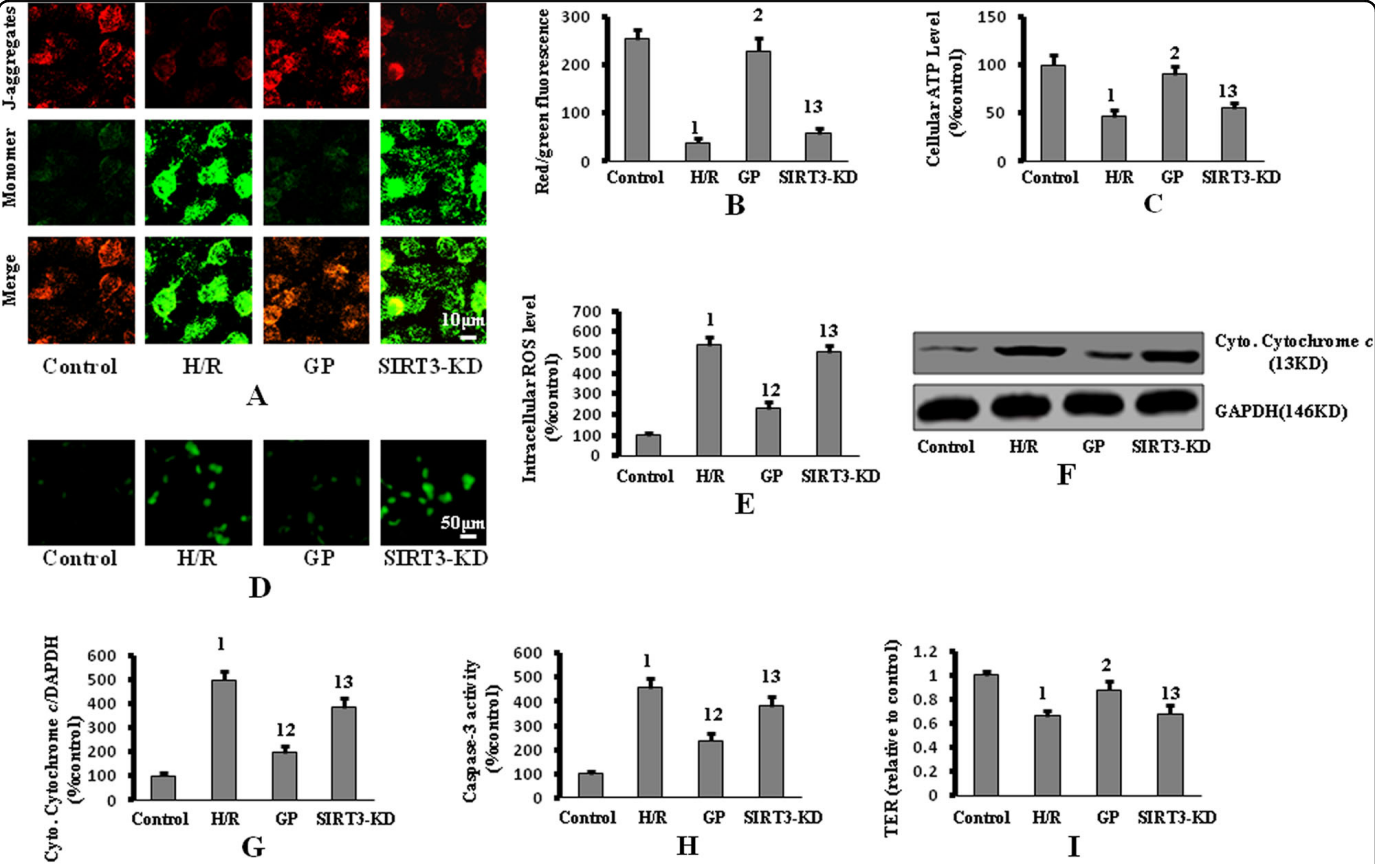
Silencing of SIRT3 reversed the protective effect of GP against H/R-induced IAS activation. **a** Intracellular red and green fluorescence of JC-1 was determined by fluorescent inverted microscopy (×400 magnification). **b** The intracellular red and green fluorescence of JC-1 was measured by flow cytometry. **c** Intracellular ATP levels were determined by a luciferase-based assay. **d** Intracellular ROS levels were determined by DCFH-DA. **e** ROS levels were quantified by an automatic microplate reader. **f** Cytoplasmic cytochrome *c* was measured by western blot. **g** Quantification of cytoplasmic cytochrome *c* protein expression by densitometry. **h** Caspase-3 activity was measured using a caspase-3 fluorometric assay kit. **i** Monolayer hyperpermeability was examined by detecting the TER of the PMVEC monolayer. Data are presented as mean ± SD (*n* = 6 in each group). 1: *P* < 0.05 compared with the control group; 2: *P* < 0.05 compared with the H/R group; 3: *P* < 0.05 compared with the GP group

GP treatment up-regulated the expression of SIRT3 in H/R, which was depressed by SIRT3 silence (Fig. 3c, d). Markers of increased autophagy, which were enhanced by GP treatment (i.e., formation of GFP-LC3 punctae, up-regulation of LC3 II and Beclin-1, and down-regulation of P62), were reversed by SIRT3 knockdown (Fig. 3a–c, e–g). In addition, depolarized mitochondria, reflected by an increase in green fluorescence and the concomitant disappearance of red fluorescence, increased ROS levels, lower ATP levels, and increased cytoplasmic cytochrome *c* and caspase-3 activity, were detected in SIRT3-knockdown cells compared to GP-treated cells without SIRT3 knockdown that were exposed to H/R (Fig. 4). These data indicate that SIRT3 is required for GP-induced autophagy, and suggest that the protective effects of GP against IAS in H/R are mediated through SIRT3.

### Inhibition of autophagy and SIRT3 reverses the protective effects of GP against HS-induced VH

To confirm the involvement of SIRT3/autophagy in mediating the protective effects of GP against HS-induced VH in vivo, 3MA and 3-TYP were used inhibit autophagy and SIRT3, respectively. Rats were divided into five groups: control group (rats pretreated with vehicle were operated on but no hemorrhage); HS group (rats pretreated with vehicle then subjected to HS and resuscitated); GP group (rats pretreated with 5 mg/kg GP were then subjected to HS and resuscitated); 3MA group (rats pretreated with 10 mg/kg 3MA were then subjected to HS and resuscitated); 3-TYP group (rats pretreated with 5 mg/kg 3-TYP were then subjected to HS and resuscitated).

**Fig. 5:**
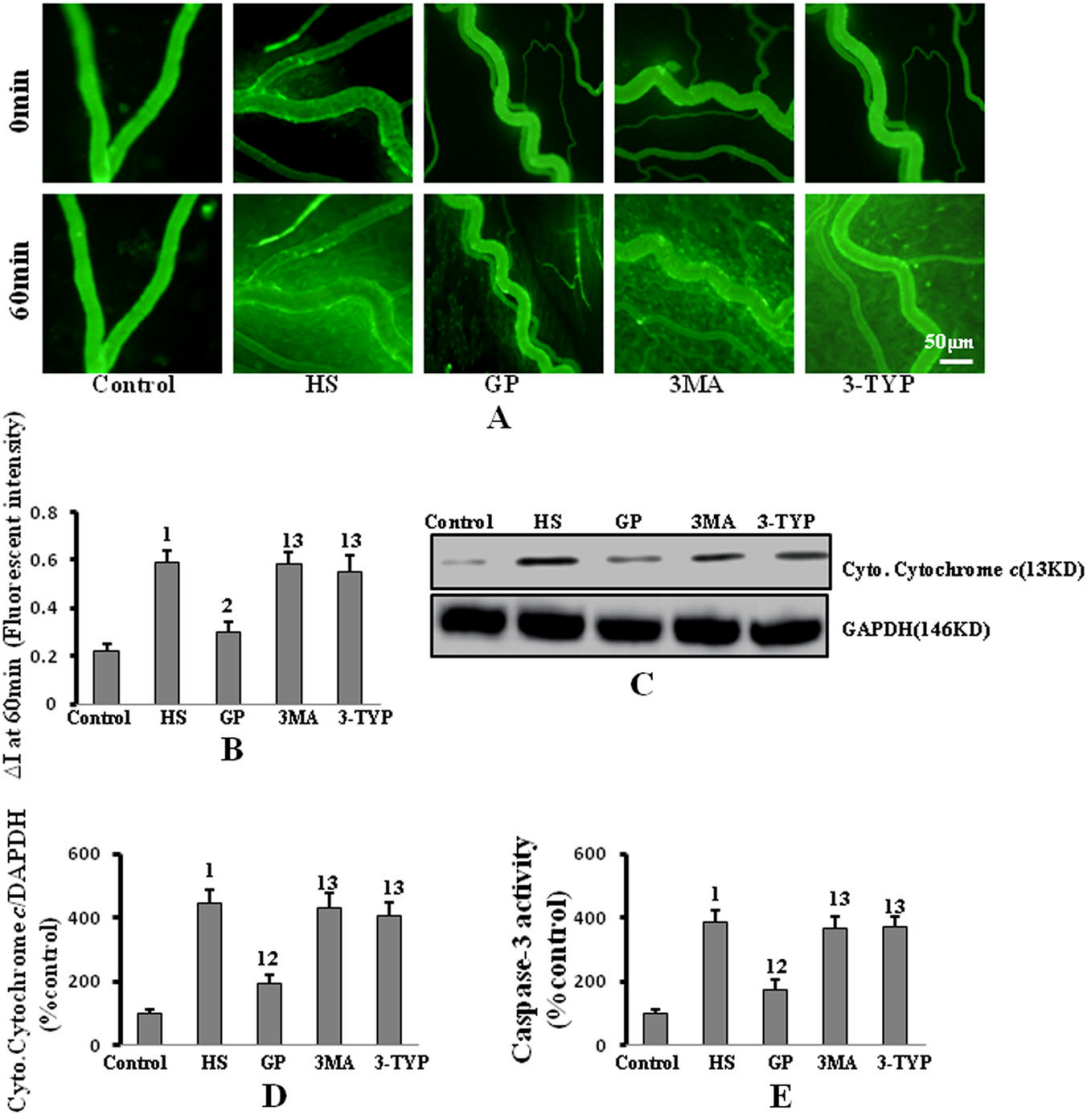
The protective effect of GP against HS-induced VH was reversed by inhibition of autophagy and SIRT3. **a** Mesenteric postcapillary venules were examined for changes in permeability by intravital microscopy. Images of mesenteric postcapillary venules at 10 and 60 min are shown (×100 magnification). **b** Vascular permeability is expressed as change in fluorescent intensity (Δ*I*) inside the vessel compared with the intensity outside the vessel. **c** Cytoplasmic cytochrome *c* in mesenteric microvasculature was measured by western blot. **d** Quantification of cytoplasmic cytochrome *c* protein expression by densitometry. **e** Caspase-3 activity in mesenteric microvasculature was measured using a caspase-3 fluorometric assay kit. Data are presented as mean ± SD (*n* = 6 in each group). 1: *P* < 0.05 compared with the control group; 2: *P* < 0.05 compared with the HS group; 3: *P* < 0.05 compared with the GP group

Changes in fluorescein isothiocyanate conjugate-bovine serum albumin (FITC-BSA) extravasation into the extravascular space indicated changes in vascular permeability. HS-induced VH in rats is indicated by a marked increase in FITC-BSA extravasation into the extravascular space, as compared to the control group. Rats treated with GP showed significantly lower FITC-BSA extravasation than the HS group (Fig. 5b). However, FITC-BSA extravasation was increased in the 3MA and 3-TYP treatment groups compared to the GP group, which indicated that inhibition of SIRT3 or autophagy mitigated the protective effects of GP treatment against HS-induced VH. A series of images of rat mesenteries obtained from the same microscopic fields at 10 and 60 min post-shock are presented in Figure 5a. At 10 min, minimal extravasation of FITC-BSA into the extravascular space was observed in all groups. However, at 60 min, markedly higher FITC-BSA extravasation was observed in the HS group compared to the control group; the extent of FITC-BSA extravasation was reduced by GP treatment. Additionally, markedly higher FITC-BSA extravasation was observed in the 3MA and 3-TYP treatment groups compared to the GP treatment group.

**Fig. 6:**
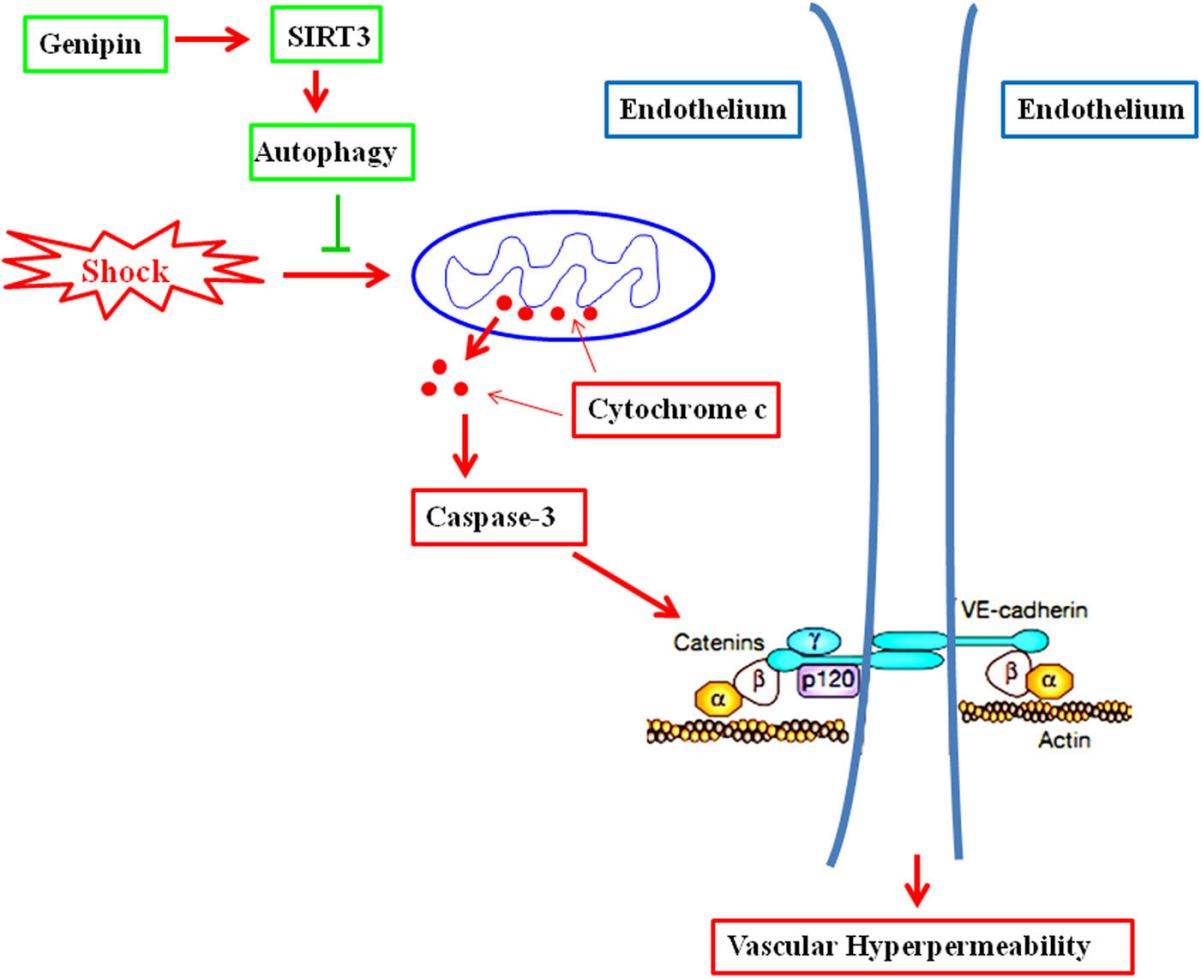
GP protects against vascular hyperpermeability by up-regulation of SIRT3 and autophagy following HS. HS-induced IAS activation ultimately leads to caspase-3 activation, inducing caspase-3 cleavage of β-catenin, which regulates VE-cadherin-mediated cell–cell adhesion in endothelial cells; this cleavage leads to vascular hyperpermeability. GP is likely to activate SIRT3, inducing the up-regulation of autophagy, which prevents activation of IAS and vascular hyperpermeability. GP genipin, HS hemorrhagic shock, IAS intrinsic apoptotic signaling, SIRT3 silent information regulator 3

In addition, increased cytoplasmic cytochrome *c* and caspase-3 activity were detected in the HS group compared to the control group (Fig. 5c–e). Furthermore, decreased cytoplasmic cytochrome *c* and caspase-3 activity were observed in rats pretreated with GP; this attenuation of apoptosis was reversed by treatment with 3MA and 3-TYP (Fig. 6). These data further confirm that GP protects against HS-induced VH by up-regulation of SIRT3 and autophagy.

## Discussion

In the present study, we demonstrated that the protective effects of GP against HS-induced VH are mediated via activation of autophagy. Our findings also highlight the potential of SIRT3 as a facilitator of GP-induced autophagy (Fig. 6).

In a previous study, we reported that IAS plays an important role in VH^6,13^. In addition, GP has been demonstrated to ameliorate MD and apoptosis^14–16^. In the present study, we hypothesized that GP inhibits HS-induced IAS and VH. To investigate the protective effects of GP against HS-induced VH, PMVECs were exposed to H/R, and the endothelial TER was evaluated. We found that GP attenuated the decrease in endothelial TER induced by H/R exposure. IAS is mediated by mitochondrial activation, resulting in the subsequent release of cytochrome *c* and second mitochondrial-derived activator of caspases, and ultimately leading to caspase-3 activation^17,18^. Caspase-3 activation results in the cleavage of various cell adherens proteins, including β-catenin, which regulate VE-cadherin-mediated cell–cell adhesion in endothelial cells;^19^ this cleavage may in turn lead to micro-VH^6,13^. In the present study, we observed activation of IAS following HS, as evidenced by MD, and indicated by collapsed MMP, lowered ATP levels, and increased cytoplasmic cytochrome *c* and caspase-3 activity. However, GP pretreatment significantly inhibited HS-induced IAS, which indicated that GP improves HS-induced VH by inhibition of IAS.

Autophagy is a regulated pathway that facilitates turnover of cellular organelles and long-lived proteins, and can promote cell survival under various stress conditions^6,13,22^. Recently, a growing body of evidence has indicated the protective effects of autophagy against endothelial hyperpermeability^4,5^. In addition, the pharmacological effects of GP in the regulation of autophagy have been demonstrated^20^. Here we report that GP up-regulated HS-induced autophagy, as evidenced by increased GFP-LC3 punctae, up-regulation of LC3 II and Beclin-1, and down-regulation of P62; this is consistent with previous reports^20^. Furthermore, we used 3MA, a specific inhibitor of autophagy, to evaluate the role of autophagy in GP-induced amelioration of VH. Interestingly, treatment with 3MA together with GP resulted in a reversal of the improvement in monolayer permeability, compared with GP treatment alone. Moreover, 3MA also reduced the protective effects of GP against HS-induced IAS. These results indicate that autophagy may protect against HS-induced VH by inhibiting IAS, and suggest that GP treatment can enhance autophagy.

Although the effects of regulation of autophagy by GP have been reported, the specific mechanism remains obscure. SIRT3 is involved in various physiological processes^21,22^, and a relationship between SIRT3 and autophagy has been reported;^10,23^ for example, Sirt3 promotes autophagy through the deacetylation of FoxO1^11^. However, it is unclear whether SIRT3 influences GP-induced autophagy. In this study, we found that silencing of SIRT3 suppressed GP-induced autophagy, as indicated by decreased formation of GFP-LC3 punctae, reduced expression of LC3 II and Beclin-1, and increased expression of P62 in GP-treated cells transfected with SIRT3 siRNA. We also found that suppression of SIRT3 reversed GP-induced improvement of IAS and monolayer hyperpermeability. Our results indicate the involvement of SIRT3 in the regulation of autophagy and endothelial barrier dysfunction by GP.

To confirm the protective effects of GP against HS-induced VH, we used intravital microscopy to characterize changes in fluorescein-labeled protein transport in the rat mesenteric venula. We found that pretreatment with GP significantly reduced HS-induced extravasation of FITC-BSA into the extravascular space. In addition, increased extravasation of FITC-BSA was observed in rats treated with GP in parallel with 3MA or 3-TYP, indicating that inhibitions of autophagy and SIRT3 mitigate the protective effects of GP against VH in vivo.

These findings suggest that GP protects against IAS and VH following HS via SIRT3 and autophagy, and indicate that the SIRT3-activating and autophagy-inducing functions of GP merit further exploration as potential therapy for VH.

## Materials and methods

### Reagents and antibodies

MitoProbe^TM^ JC-1, DCFH-DA and MitoTracker^TM^ (Thermo-Fisher, Carlsbad, CA, USA) were purchased from Molecular Probes (Invitrogen, CA, USA). The CellTiter-Glo^®^ assay was supplied from Promega Corp. (Madison, WI, USA). A mitochondrial/cytosolic protein extraction kit was purchased from BestBio Co. (Beijing, China). Antibodies against P62, LC3, Beclin-1, Sirt3, and GAPDH were obtained from Abcam (Cambridge, UK). The SIRT3-targeting RNAi kit was purchased from Santa Cruz Biotechnology (Santa Cruz, TX, USA). Rat PMVECs were obtained from Guangzhou Cellcook Biotech Co., Ltd (Guangzhou, China). The Cell Count Kit-8 (CCK-8) was purchased from Dojindo Co. (Shanghai, China). GP, FITC-albumin, and other chemicals were purchased from Sigma-Aldrich (Saint Louis, MO, USA).

### Cell culture and H/R

Rat PMVECs were maintained in DMEM/F12 containing 10% fetal bovine serum at 37 °C in a humidified atmosphere (95% air, 5% CO_2_). Before experimentation, confluent cell monolayers formed on culture dishes, microporous transwell filter inserts, or glass coverslips within 24 h. As previously described^24^, cells were treated with hypoxia for 48 h (5% CO_2_, 1% O_2_, and 94% N_2_), followed by 2 h of reoxygenation (5% CO_2_, 21% O_2_, and 74% N_2_) to establish H/R in vitro*.* Various concentrations (20, 50, and 100 μM) of GP were added to cells 2 h before H/R. Cells were exposed to H/R and cell viability was determined using a CCK-8 kit, in accordance with manufacturer's instructions. GP (50 μM) was selected as the “ideal” concentration for in vitro experiments.

### TER measurement

The TER of PMVECs monolayers was determined using an STX2 electrode and EVOM2 meter, according to the manufacturer’s instructions (World Precision Instruments, Sarasota, FL, USA). PMVECs were seeded at 1 × 10^5^ cells per cm^2^ on fibronectin-coated 6.5 mm transwell filters (0.4 mm pore size) and were allowed to grow to confluence. After H/R treatment, the resistance values of multiple transwell inserts for an experimental group were measured sequentially, and the mean was expressed in the common unit (Ω cm^2^) after subtraction of the blank cell-free filter.

### Experimental animals

The procedures used in this study and the handling of study animals adhered to the National Institutes of Health guidelines on the use of experimental animals. The experimental protocol was approved by the Committee on Research Animal Use of South Medical University. Adult male Sprague–Dawley rats, weighing 180–220 g, were purchased from the Experimental Animal Center at South Medical University and were allowed to acclimatize for 1 week before use. Animals had ad libitum access to food and water.

### Hemorrhagic shock

Rats were anesthetized with an intramuscular injection of sodium pentobarbital (30 mg/kg). Mean arterial blood pressure (MAP) was continuously measured via a PE-50 cannula in the femoral artery. A second cannula in the femoral vein was used to administer drugs and blood, and a third cannula placed in another femoral artery was used for blood withdrawal. Rats received treatment with GP or 3MA and 3-TYP 10 min before the start of the experiments. To produce HS, as previously described (16), blood was withdrawn from the cannula in the third femoral artery into a syringe containing diluted heparin solution (125 U/mL) in order to dramatically reduce the MAP to 40–45 mm Hg within 10 min; this MAP was maintained for 120 min. After the shock period, the shed blood twice the volume of normal saline was reinfused into the animal to maintain the MAP at ≥90 mm Hg.

### Vascular permeability measurement

Vascular permeability was measured as previously described^25,26^. Briefly, the rats were placed on a Plexiglas^®^ platform mounted on an intravital upright microscope (ECLIPSEFN1; Nikon, Japan). A midline laparotomy was performed, and a section of the mesentery from the proximal ileum was draped over the optical stage for observation (×100 magnification). After intravenous injection of 50 mg/kg FITC-BSA, postcapillary venules ranging from 20 to 50 μm in diameter were visualized using the intravital microscope.

The transvascular flux of plasma proteins was assessed by comparing the relative changes in the fluorescence intensity of FITC-BSA between the intravascular and extravascular space. The following formula was used to calculate changes in integrated optical intensity: Δ*I* = 1 − (*I*_i_ − *I*_o_)/*I*_i_, where Δ*I* is the change in light intensity, I_i_ is the light intensity inside the vessel, and *I*_o_ is the light intensity outside the vessel. Parameters were recorded at 60 min followed HS. Rats were pretreated with GP at various doses (2.5, 5, and 7.5 mg/kg), followed by HS and measurement of vascular permeability. Based on the results of measurement in vascular permeability, 5 mg/kg was selected for in vivo GP treatment.

### siRNA transfection

siRNA targeting SIRT3 was purchased from Santa Cruz Biotechnology. Scrambled non-targeting siRNA was used as a control. Transfection of siRNA was performed according to the manufacturer’s protocol. Briefly, cells in exponential growth phase were plated in six-well tissue culture plates at 1 × 10^5^ cells per well, grown for 24 h, and then transfected with siRNA using Oligofectamine^TM^ and Opti- MEM^TM^ (Thermo-Fisher Scientific, USA) reduced serum medium.

### Detection of autophagy using GFP-LC3

The GFP-LC3 fusion protein provides a useful indicator of initiation of autophagy through the evaluation of LC3 dots, or “punctae”. When autophagy occurs, GFP-LC3 foci redistribute from a diffuse pattern to a punctate cytoplasmic pattern (GFP-LC3 punctae), and the percentage of cells with GFP-LC3 punctae increases.

Cells were transfected with ptfLC3 (Addgene, plasmid #21074), a highly specific fluorescent marker of autophagy, to measure levels of autophagy. Lipofectamine^®^ 3000 Reagent (Thermo-Fisher Scientific, USA) was used to transfect cells. After the treatment and H/R exposure, the cellular localization of GFP-LC3 was visualized using a confocal microscope (LSM780; Zeiss Microsystems, Jena, Germany).

### Measurement of MMP

The MMP was determined using the potential-sensitive fluorescent dye JC-1. Cells were treated and subjected to H/R. JC-1 (5 μmol/L) was loaded onto cells for 15 min at 37 °C. The results were visualized using an inverted fluorescent microscope (Nikon, Ti-E Live Cell Imaging System, Japan).

### Measurement of cellular ATP

Intracellular ATP was determined by a luciferase-based assay (CellTiter-Glo^®^, Promega, Madison, WI, USA), according to the manufacturer’s recommendation. Cells were treated and subjected to H/R. After H/R, 100 μL CellTiter-Glo^®^ reagent was added to 100 μL of cell suspension containing 10,000 cells in each well of a standard opaque-walled 96-well plate. After that, the plates were allowed to incubate at room temperature for 10 min and the luminescence was recorded in an automatic microplate reader (SpectraMax M5; Molecular Devices, Sunnyvale, CA, USA). Measurement of intracellular reactive oxygen species (ROS) levels Intracellular ROS levels were assessed using DCFH-DA probe. Cells were treated with DCFH-DA (10μM), following sham/burn serum, for 20 mins at 37 °C. After incubation, the cells were observed using a fluorescence microscope (ECLIPSE FNl, Nikon, Tokyo, Japan) and analyzed using an automatic microplate reader (SpectraMax M5; Molecular Devices, Sunnyvale, CA, USA).

### Caspase-3 activity measurement

The activity of caspase-3 in vitro and in vivo was measured using the caspase-3/CPP32 fluorometric assay kit in keeping with the manufacturer’s instructions.

### Western blotting

Rat mesenteric microvasculature was dissected from rats 60 min after the rats were exposed to HS for 120 min. PMVECs were exposed to H/R and were collected. Samples of mesenteric microvasculature and PMVECs cells were lysed and homogenized to obtain total protein. Cytoplasmic protein was isolated from mesenteric microvasculature and PMVECs using a mitochondrial/cytosolic protein extraction kit according to the manufacturer’s instructions.

Total protein and cytoplasmic protein were extracted and mixed with 5× sodium dodecyl sulfate (SDS) sample buffer. Samples were separated by SDS-polyacrylamide gel electrophoresis using 8–12% acrylamide gels and transferred to PVDF membranes. After incubation with primary antibodies against P62, Beclin-1, LC3, cytochrome *c*, and SIRT3, membranes were incubated with the appropriate secondary antibodies. Protein bands were visualized and detected using chemiluminescence detection reagents.

### Statistical analysis

All data are presented as means ± SD. Differences between groups were determined using one-way analysis of variance with the least significant multiple-comparison test and Student’s *t* test, when appropriate. Values were considered significant when *P* < 0.05.
